# Remarkable colorimetric sensing behavior of pyrazole-based chemosensor towards Cu(ii) ion detection: synthesis, characterization and theoretical investigations†

**DOI:** 10.1039/c8ra02905a

**Published:** 2018-05-17

**Authors:** Nagaraj Nayak, Kollur Shiva Prasad, Renjith Raveendran Pillai, Stevan Armaković, Sanja J. Armaković

**Affiliations:** Chemistry Group, Manipal Centre for Natural Sciences, Manipal Academy of Higher Education (MAHE) Manipal Karnataka – 576 104 India shivachemist@gmail.com; Department of Physics, TKM College of Arts and Science Karicode Kollam Kerala India; University of Novi Sad, Faculty of Sciences, Department of Physics Trg D. Obradovića 4 21000 Novi Sad Serbia; University of Novi Sad, Faculty of Sciences, Department of Chemistry, Biochemistry and Environmental Protection Trg D. Obradovića 3 21000 Novi Sad Serbia

## Abstract

We report the synthesis of a new imine based ligand, 3-((3-methoxybenzylidene)amino)-1*H*-pyrazol-5-ol (HL) and its Cu(ii) complexes in 2 : 1 (HL : metal) and 1 : 1 : 1 (HL : metal : HQ) stoichiometric ratio using 8-hydroxyquinoline (HQ) as an additional bidentate ligand. The synthesized ligand (HL) and its Cu(ii) complexes (1 and 2) are structurally characterized using FT-IR, electronic absorption and emission, NMR and MS techniques. Furthermore, the complexation of Cu^2+^ with HL leads to the immediate formation of brown colour solution which indicates that HL can act as simple colorimetric sensor for Cu^2+^ ions. We further investigated that the sensor could selectively bind to the Cu^2+^ ions even in the presence of competitive ions such as Mn^2+^, Fe^2+^, Co^2+^, Ni^2+^, Zn^2+^, Ag^+^ and Na^+^ ions in aqueous solutions which was studied by electronic absorption spectroscopy. The HL ligand has been investigated for its reactive properties by density functional theory (DFT) calculations. Quantum molecular descriptors describing local reactive properties have been calculated in order to identify the most reactive molecule sites of title compounds. DFT calculations encompassed molecular electrostatic potential (MEP), local average ionization energies (ALIE), Fukui functions and bond dissociation energies for hydrogen abstraction (H-BDE).

## Abstract 2

A novel chemosensor for Cu^2+^ ion detection with LOD 1.6 μM.

## Graphical abstract

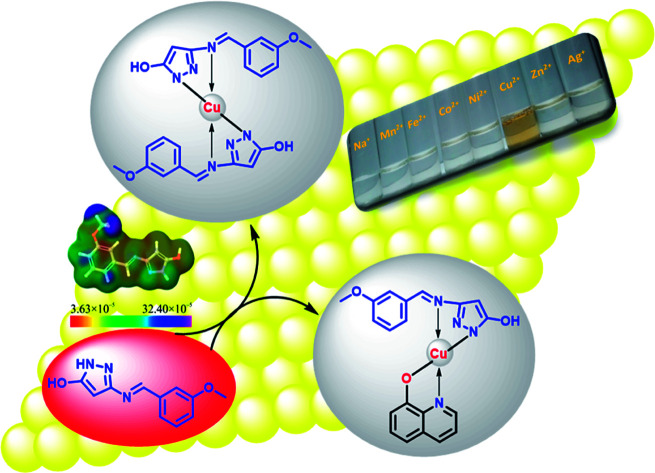

## Introduction

Cost effective colorimetric chemical sensors with simple design have attracted a tremendous interest in monitoring many environmental and biologically relevant species such as metal ions over the past few years.^1–4^ When compared with electrochemical and fluorescent chemosensors, the colorimetric methods are more promising for the detection of metal ions especially in the field because of naked eye detection. The Cu^2+^ ion is one of the most important divalent metal ions in the human body.^5^ The pivotal role of Cu^2+^ ion in most living organisms includes its key role in metabolism and immune system.^6^ On the other hand, the presence of excess of Cu^2+^ ion concentration in the organisms may cause lethal issues.^7,8^ Several methods such as atomic absorption spectroscopy,^9^ inductively coupled plasma mass spectrometry (ICP-MS),^10^ inductively coupled plasma atomic emission spectrometry (ICP-AES)^11^ are available for the determination of metal ions with some major disadvantages like sample destruction, high cost, necessary pretreatment procedures *etc.* Furthermore, there are many reports about the synthesis and use of chemosensors as metal ion probes with low to moderate detection ability.^12–15^ For instance, Goswami *et al.*, reported a highly selective colorimetric as well as fluorimetric sensor which could detect Cu^2+^ ions at a concentration as low as 5 μM.^16^ Kim *et al.*, have recently shown a naked eye chemosensor for the simultaneous detection of copper and iron ions,^17^ with Cu^2+^ ions detection limit 2.9 μM, which is well below the recommended value (31.5 μM) of the World Health Organization in drinking water. Duraisamy *et al.*, have developed a thiophene-based ligand for the detection of heavy metal ions and reported their computational studies and application in living cells.^18^ In addition, there are several recent reports on graphene oxide and silica-based materials used as probes for the detection of ions/organic molecules.^19–21^ Hence, the design and synthesis of low cost, non-natural sensors for Cu^2+^ ion detection is important and being continuous interest for researchers as it is a challenging task in terms of obtaining selective and specific sensor.

Even though there are many reports available in the area of colorimetric as well as fluorescent sensors for sensing Cu^2+^ ions,^22,23^ those colorimetric sensors which are prepared by cost effective synthetic methods are relatively seldom.^24^ Further, those colorimetric sensors which can respond at extremely low concentration of analyte are in high demand in the analytical and scientific areas, since, such low concentration of analyte(s) can be presently detected by expensive instrumentations and synthetically complex molecular systems, like porphyrins.^25^ Schiff base ligands have been proven to be the best ligands for coordination complexes because of their directionality towards metal coordination and the strength of such bond. Moreover, several of the previously reported sensors for Cu^2+^ ions simultaneously sense other competitive ions.^17^ Thus, design and construction of a cost-effective, simple and highly specific chemosensor that can be used as a colorimetric sensor for metal ion detection is of prime interest and greatest challenge as well.

Considerable attention has been extended over a decade to the heterocycles containing more than one hetero-atom, namely, imidazole, pyrazole, pyrazine, pyrimidine *etc.* with potential donor sites to form various metal coordination complexes. In these type of interaction, the π-electrons on the heteroatom actively participate in the formation of stable metal–ligand (M–L) bonds during metallation.^26^ Since, pyrazole and its derivatives act as stronger π-donor and weaker π-acceptor than the six membered heterocyclic analogues they behave as hard donors during complexation with metal ion. The multidimensional applications of pyrazole derivatives and their coordination compounds have witnessed in diverse disciplines of sciences.^27–30^ The electronic behaviour of pyrazole based ligands and their metal complexes are of prime interest because of typical behaviour of pyrazole backbone in stabilizing the electronic and optical properties of metal complexes.^31–33^ DFT calculations have been widely accepted as indispensable computational tools for prediction of reactive properties of new molecules, complexes and nanomaterials.^34–41^

In the present study, we have synthesized and characterized HL and its Cu(ii) complexes (1 and 2). Further, the HL is used as colorimetric sensor for the detection of Cu^2+^ ion in the presence of various competing ions. We also report the sensitivity of HL in Cu^2+^ ion detection, and is found to be 1.6 μM which is much lower than the recommended value (31.5 μM) of the World Health Organization for drinking water. We also performed the calculations of quantum-molecular descriptors that describe local reactivity included molecular electrostatic potential (MEP) and local average ionization energies (ALIE) obtained by mapping of their values to the electron density surface. In order to better understand fundamental properties of HL ligand, additionally Fukui functions and bond dissociation energies for hydrogen abstraction (H-BDE) have been investigated as well.

## Experimental section

### Materials and methods

3-Amino-5-hydroxypyrazole and *m*-anisaldehyde were purchased from Sigma Aldrich and were used as received. The solvents were purchased from Merck and used without further purification. The completion of reaction was monitored by thin layer chromatography (TLC) performed on pre-coated silica-gel plates (Merck, India) and spots were visualized by UV irradiation. FT-IR spectral measurements were recorded on Perkin-Elmer spectrometer version 10.03.09 (KBr pellet technique). ^1^H and ^13^C NMR were obtained using a 500 MHz Bruker Avance DPX spectrometer with TMS as internal standard. HR-ESI-MS analysis was performed on a Thermo Scientific Exactive mass spectrometer by electrospray ionization technique. The electronic absorption spectra were recorded using UV1800 spectrophotometer (Shimadzu). Steady-state fluorescence experiments were performed with a SPEX Fluorolog F112X spectrofluorometer by using optically dilute solutions.

### Computational details

DFT calculations on HL ligand and its corresponding Cu(ii) complexes have been performed with Jaguar^42–44^ program. Prior to DFT calculations all possible conformers of HL were generated with MacroModel^38^ program using OPLS3 (ref. 45–47) force field. Obtained conformers were then geometrically optimized within the framework of DFT with B3LYP^48^ exchange-correlation functional. The lowest energy conformation was checked for its ground state by vibrational analysis which yielded only positive frequencies. For all DFT calculations a LACVP(d,p) basis set was employed. Maestro^49^ program was used for preparation of input files and visualization of results. Maestro, MacroModel and Jaguar programs were used as incorporated in Schrödinger Materials Science Suite 2017-4.^50^

### Chemical synthesis

#### Synthesis of (*E*)-3-((3-methoxybenzylidene)amino)-1*H*-pyrazol-5-ol (HL)

**Scheme 1:**
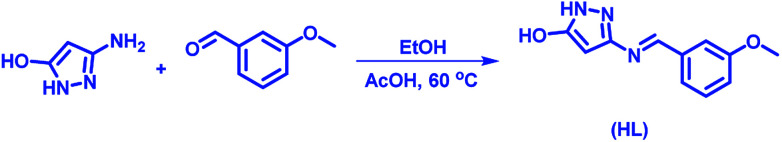
Synthesis of 3-((3-methoxybenzylidene)amino)-1*H*-pyrazol-5-ol (HL).

The title compound HL was synthesized as per Scheme 1. To a solution of 3-amino-1*H*-pyrazol-5-ol (100 mg, 1 mmol) in 20 ml of ethanol, a solution of *m*-anisaldehyde (137.8 mg, 117 μl, 1 mmol) in ethanol (15 ml) was added with continuous stirring and the reaction mixture was refluxed for 10 h in the presence of catalytic amount of acetic acid. The solid product formed was washed with ethanol and recrystallized with hot ethanol to obtain desired product in pure form.

Yield: 197 mg (91%). FT-IR (KBr pellet, cm^−1^) 3164–3325 (broad, –OH and –NH), 2991 (aromatic –CH), 2836 (–OCH_3_), 1592 (C=N), 1269 (C–O), 1039 (C–N). ^1^H NMR (DMSO-d_6_): *δ* 9.1 (s, 1H), 9.09 (s, 2H), 7.2 (t, 1H, *J* = 5 Hz), 7.06 (t, 2H, *J* = 5 Hz), 6.9 (d, 1H, *J* = 5 Hz), 6.8 (d, 1H, *J* = 5 Hz), 6.7 (t, 1H, *J* = 5 Hz), 6.6 (d, 1H); 5.3 (s, 1H), 6.9 (d, 1H, *J* = 5 Hz), 4.5 (s, 1H), 4.1 (s, 1H), 3.7 (s, 3H), 3.6 (s, 3H). ^13^C NMR (DMSO-d_6_, ppm): *δ* 171.6, 158.1, 157.2, 156.2, 136.8, 128.2, 126.9, 120.8, 120.4, 114.3, 113.4, 112.2, 110.9, 81.6, 60, 56.3, 54.3 and 43.06 (ESI).† ESI-MS calculated for C_11_H_11_N_3_O_2_ [M + H]^+^: 218.09 and found is 218.09 (monomer mass) and calculated is 435.17 and found is 435.177 (dimer mass).

#### Synthesis of Cu(HL)_2_ complex (1)

The title complex was synthesized by adding two equivalents of methanolic solution of ligand (108.5 mg, 0.5 mmol) to a continuously stirred methanolic solution of Cu(CH_3_COO)_2_. H_2_O (0.25 mmol, 49.9 mg). Immediate change in colour to dark brown has been observed after which the solution was refluxed at 60 °C for 12 h. The cooled reaction mixture was filtered and the dark brown solid obtained was repeatedly washed with methanol to obtain the pure product. Yield of the complex = 56%. FT-IR (KBr pellet, cm^−1^) = 3160–3420 (broad, –OH and –NH), 2951 (aromatic –CH), 2841 (–CH_3_), 1609 (C=N), 1258 (C–O) and 1039 (C–N). ESI-MS calculated for C_22_H_22_N_6_O_4_Cu [M]^+^: 497.17 and found is 497.100 (ESI).†

#### Synthesis of Cu(HL)(HQ) complex (2)

54.2 mg (0.25 mmol) of Schiff base ligand and 36.27 mg (0.25 mmol) of 8-hydroxy quinoline were dissolved in 25 ml of dry methanol. To this clear solution, 49.9 mg (0.25 mmol) of methanolic solution of Cu(CH_3_COO)_2_. H_2_O was added and the mixture was refluxed for 12 h at 60 °C. The cooled reaction mixture was filtered and the dark brown solid obtained was repeatedly washed with methanol to obtain the pure product. Yield: 34%; FT-IR (KBr pellet, cm^−1^): 3106–3446 (broad, –OH and –NH), 2974 (aromatic –CH), 2830 (–CH_3_), 1693 (C=N), 1597, 1482, 1378 (C–O) and 1114 (C–N). ESI-MS calculated for C_20_H_16_N_4_O_3_Cu [M]^+^: 423.05 and found is [M + K]^+^: 462.81 (ESI).†

## Results and discussion

**Fig. 1:**
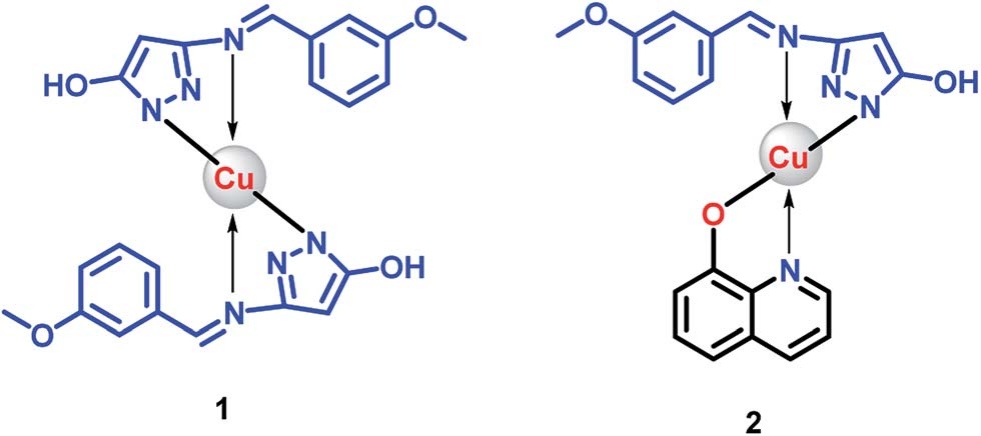
Proposed structures for Cu(ii) complexes.

The ligand (HL) was obtained according to the synthetic route showed in Scheme 1. The spectroscopic data provided information in deducing molecular structure of compounds. The proposed structures of 1 and 2 are showed in Fig. 1.

**Fig. 2:**
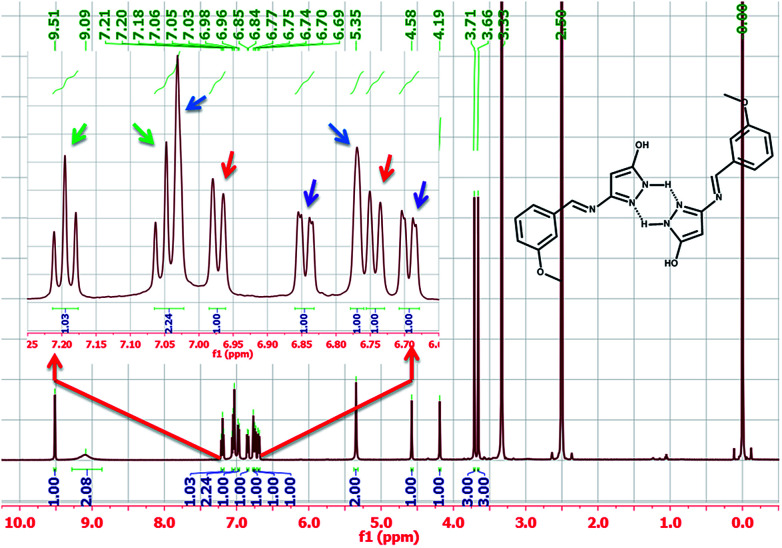
The proton NMR spectrum of HL ligand.

One of the surprising observations is that the ligand existed in dimeric state which is evident from its ^1^H NMR and ESI-MS spectra. (The repeated purification of the ligand resulted in similar NMR). The formation of stable 6 membered ring structures because of hydrogen bonding interaction between pyrazole moieties may be the reason for dimer formation. A clear evidence for the dimer formation could be seen by analysing the NMR spectra of HL and proposed structure of dimer is shown along with NMR spectra in Fig. 2.

**Fig. 3:**
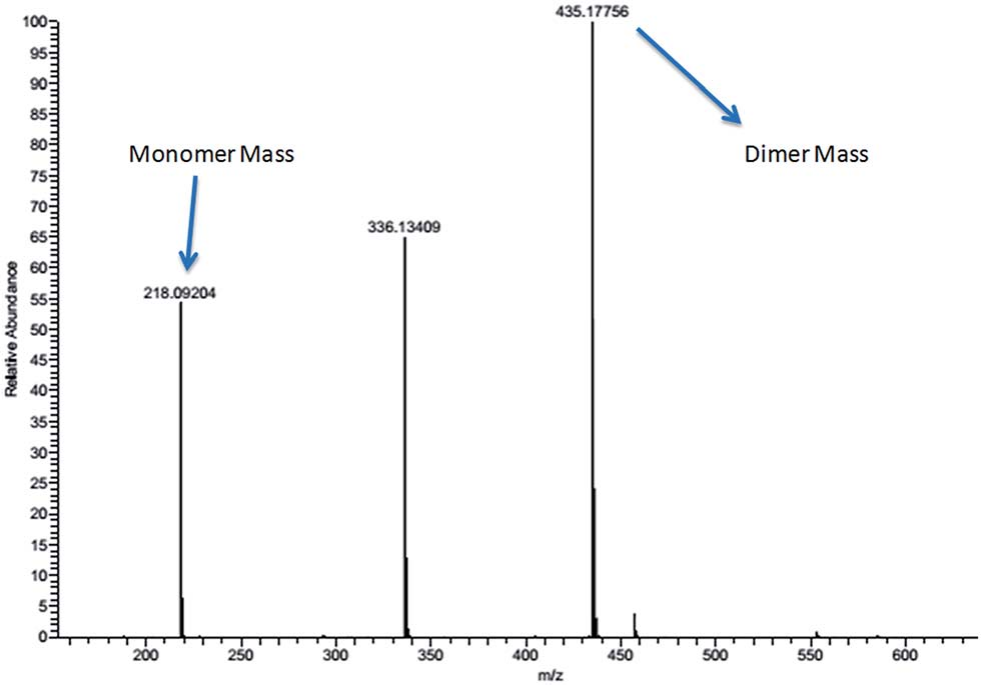
The ESI-MS spectrum of compound HL showing monomeric and dimeric species.

The aromatic peaks of the HL are shown as inset which consists of four pairs of peaks with similar integration and similar splitting nature. Surprisingly, all these peaks had an integration of one proton, suggesting that each pair of these peaks belong to same kind of protons from two different parts of dimer. The picture was more clear when we recorded the mass spectra of the HL, which even in dilute solution showed two prominent mass peaks which corresponds to monomeric and dimeric species (Fig. 3).

The IR spectra for both ligand and its copper complexes are shown in ESI.† The IR band which appeared at 1592 cm^−1^ for the free ligand (HL) is due to CH=N group, showed a considerable shift towards higher wave numbers (1609 cm^−1^ in 1 and 1693 cm^−1^ in 2) which suggests that the azomethine group is involved in the coordination to copper ion in both the complexes. Further, the peak around 3200 cm^−1^ due to –OH and –NH of ligand became further broad possibly due to the involvement of –NH group in the coordination of copper center.

**Fig. 4:**
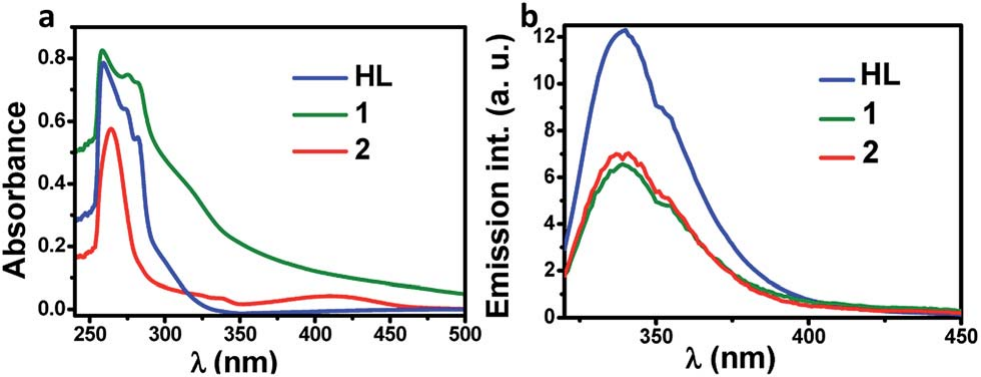
The absorption (a) and emission (b) spectra of HL and its complexes, 1 and 2 recorded in DMSO.

In order to evaluate the photophysical properties of ligand and metal complexes, the electronic absorption and emission spectra were recorded using their DMSO solution. As shown in Fig. 4, the HL showed characteristic absorption around 250 nm to 290 nm with emission extending up to 400 nm. Further, the absorption spectrum of complex 1 showed a broad peak extending upto 450 nm while the complex 2 showed a small new band near 420 nm. The emission profile of the two complexes were similar to parent ligand but the presence of metal has essentially quenched the ligands emission.

MEP surfaces are frequently used descriptors for description of local reactivity properties. This descriptor is visualized by mapping of its values to the electron density surface and is regarded as a fundamental quantity describing reactivity of molecules. MEP, *V*(*r*), is defined according to the next equation:1
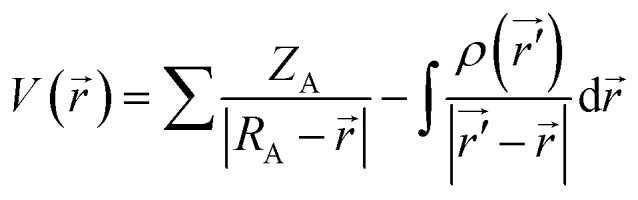


Neglecting polarization and nuclear rearrangement effects due to the presence of a unit test charge at distance *r*. In eqn (1)*ρ*(*r*′) denotes molecule's electron density, while summation is performed over all nuclei A with charge *Z*_A_ and coordinate *R*_A_. *V*(*r*) is interpreted as potential exerted at coordinate *r* by nuclei and electrons, whereas the sign of this descriptor at any point indicates whether the effects of electron or nuclei is dominant.^51,52^ Molecule sites with the lowest/highest values of MEP indicate areas where electrostatic interactions with other molecules might occur. Taking everything into account, it can be concluded that MEP surface is very useful descriptor for identification of molecule sites prone to electrophilic attacks.

However, identification of molecule sites prone to electrophilic attacks might be better performed employing the ALIE descriptor. ALIE gives the information about the energy required to remove an electron from molecule and having said that the locations of its lowest values indicate sites sensitive towards electrophilic attacks. Same as MEP, this descriptor is visualized by mapping of its values to the electron density surface. ALIE was defined as a sum of orbital energies weighted by the orbital densities,^53,54^ according to the following equation:2
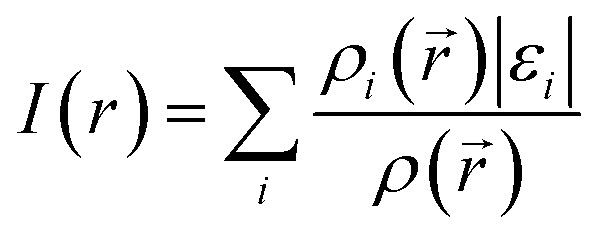
*ρ*_*i*_(*r⃑*) in eqn (2) represents the electronic density of the *i*-th molecular orbital at the point *r⃑*,*ε*_*i*_ represents the orbital energy, while *ρ*(*r⃑*) is the total electronic density function.

**Fig. 5:**
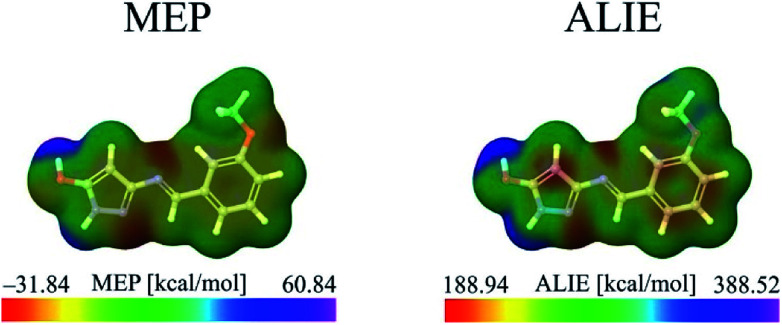
MEP and ALIE surfaces of HL ligand.

The identification of the molecule sites prone to electrophilic attacks is certainly the most efficient if both MEP and ALIE surfaces are employed, which has been provided in Fig. 5 for HL ligand.

Both MEP and ALIE surfaces recognize near vicinity of nitrogen atoms N10 and N15 to be sensitive towards the electrophilic attacks. MEP surface also recognizes near vicinity of oxygen atom O7 to be sensitive to electrophilic attacks. Aside of nitrogen atoms N10 and N15, ALIE surface recognizes several locations within five membered and six membered rings also sensitive towards electrophilic attacks. These locations are near vicinity of carbon atoms C1, C4, C5 and C12.

**Fig. 6:**
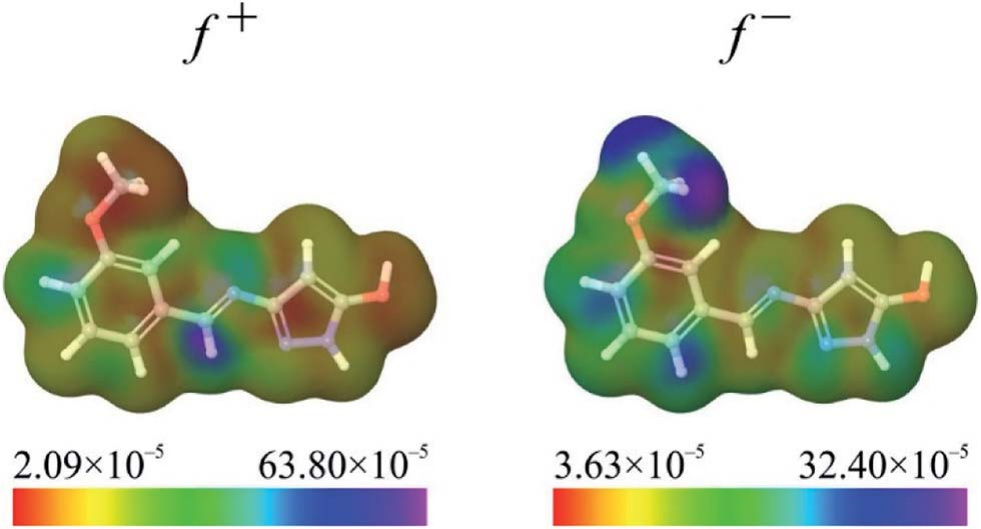
Representative Fukui functions of HL ligand.

Additionally, local reactive properties of HL ligand were investigated employing the Fukui functions as well. In Jaguar program for DFT calculations, Fukui functions are calculated in finite difference approximation according to the following equations:3
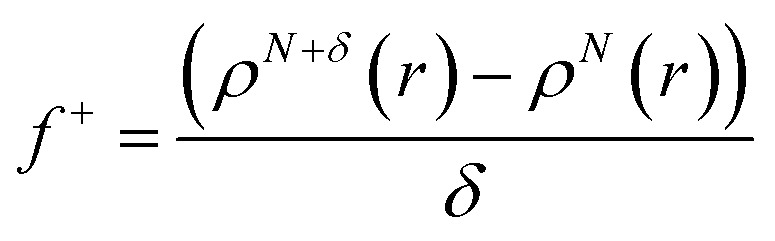
4
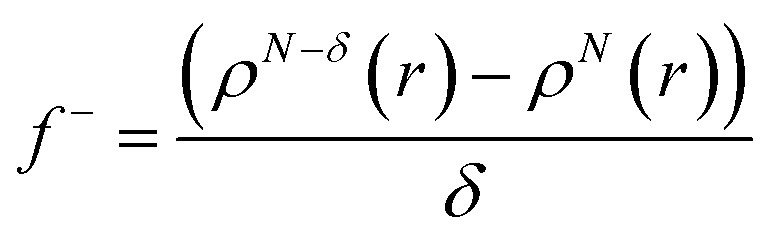
where *N* denotes the number of electrons in the reference state of the molecule and *δ* represents the fraction of electron, which is set to be 0.01.^50^ Fukui functions of HL ligand are presented in Fig. 6.

Fukui functions indicate how electron density changes with changes of the overall charge. Maximal values of Fukui *f*^+^ function, marked with purple colour in left panel of Fig. 6, indicate molecular areas where electron density increased and therefore electrophilic parts of molecule. According to these results, near vicinity of C9–H17 bond is characterized by the increase of electron density as a consequence of charge addition. Minimal values of Fukui *f*^−^ function, marked with red colour in right panel of Fig. 6, indicate molecular areas where electron density decreased, and therefore nucleophilic parts of molecule. In case of Fukui *f*^−^ function, results in Fig. 6 indicate that near vicinity of bond C1–C2 are characterized by the electron density decrease as a consequence of charge removal.

**Fig. 7:**
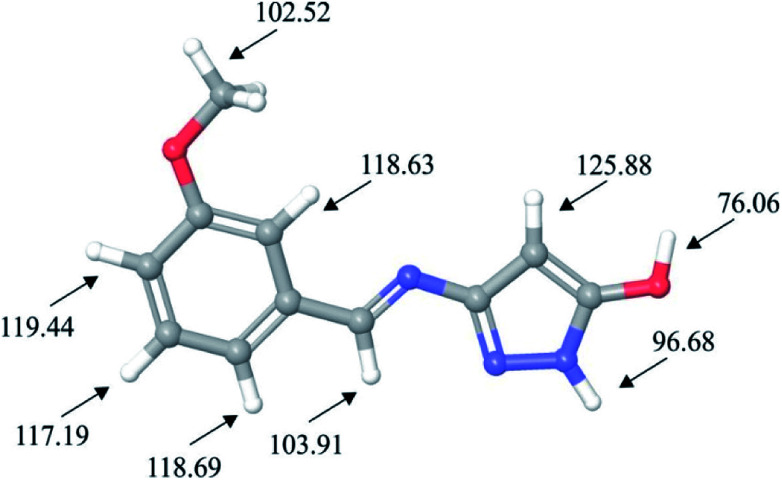
H-BDE values of HL ligand.

Taking into account the importance of oxidative reactions, in this work we have also used DFT calculations to locate molecular sites of HL ligand possible sensitive towards the autoxidation mechanism. Autoxidation mechanism is of great industrial importance and the ability to predict sensitivity of molecular structure towards it is very significant. Autoxidation is correlated with bond dissociation energies for hydrogen abstraction (H-BDE), a parameter which can be used for prediction of sensitivity of molecule towards this mechanism. Molecular sites where H-BDE takes values between 70 kcal mol^−1^ to 85 kcal mol^−1^ are considered to be highly sensitive towards autoxidation,^55,56^ while H-BDE values between 85 kcal mol^−1^ to 90 kcal mol^−1^ might indicate certain sensitivity towards autoxidation, but other influences might prevent the autoxidation.^57^ It should also be mentioned that H-BDE values lower than 70 kcal mol^−1^ are not indicating sensitivity towards autoxidation mechanism.^58,59^ H-BDE values for HL ligand have been summarized in Fig. 7.

H-BDE values provided in Fig. 7 indicate that HL ligand is highly sensitive towards autoxidation mechanism due to the fact that BDE for hydrogen abstraction of hydrogen atom H27 is 76 kcal mol^−1^. Other H-BDE values are much higher than the desired upper border level of 90 kcal mol^−1^. Second lowest H-BDE value has been calculated for hydrogen atom of pyrazole ring, but this value is almost 7 kcal mol^−1^ higher than the upper border level.

**Fig. 8:**
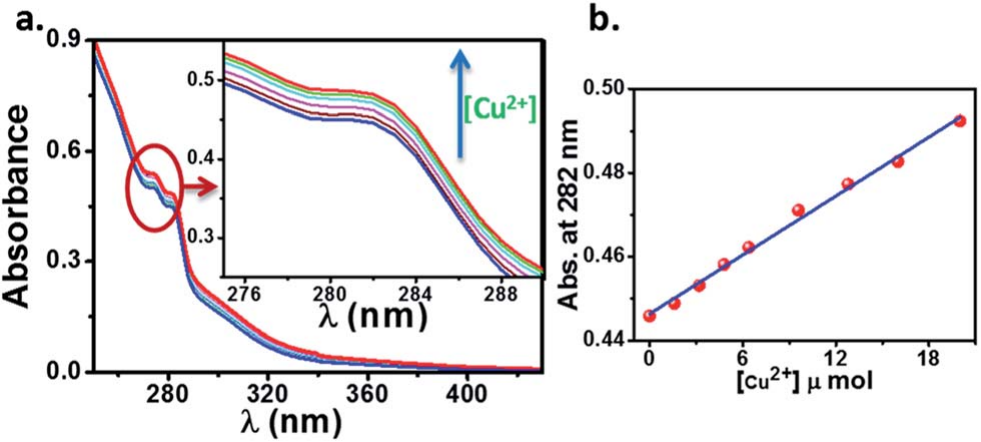
(a) The change in absorption spectrum of HL (10 μM) upon addition of aqueous Cu^2+^ solution (1.6 μM each time). (b) The concentration dependence of absorption of HL to Cu^2+^ at lower concentrations (up to 1 : 2, ligand : metal ratio).

**Fig. 9:**
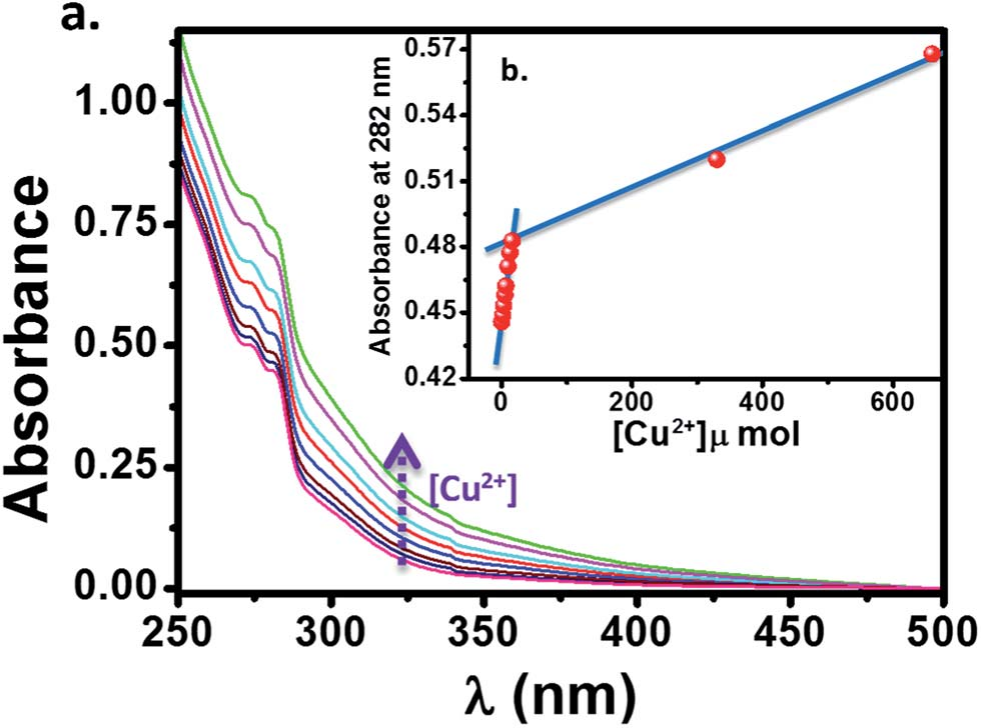
(a) The change in absorption spectrum of HL (10 μM) upon addition of aqueous Cu^2+^ solution. (b) The concentration dependence of absorption of HL to Cu^2+^ at lower concentrations (up to 1 : 2, ligand : metal ratio) and at higher concentrations (1 : 150, ligand : metal ratio).

The cation sensing of the chemosensor HL was studied by UV-visible spectroscopy in their water/DMSO (9 : 1, v/v) solutions. The HL shows two characteristic absorption bands located near 275 and 282 nm in its water/DMSO (9 : 1, v/v) solution. The change in the absorption spectrum of HL (10 μM) upon addition of aqueous Cu^2+^ solution is showed in Fig. 8. We observed an enhancement in the absorption peaks of ligand upon addition of Cu^2+^ solution with simultaneous change in color from colorless to brown. This suggests that, the ligand HL can serve as a potential candidate for “naked eye” Cu^2+^ sensor. It is to be noted that, there is a linear behaviour (Fig. 8b) in this mole ratio plot until the ratio of ligand to metal concentration is 1 : 2, after which absorption showed a sharp dip with further increase in the concentration of metal ions (Fig. 9b). This behaviour at lower concentration of Cu^2+^ can be attributed to the reaction of added Cu^2+^ with the free HL molecules which otherwise exist largely in dimeric state and thus system follows Beer–Lambert law. On the other hand, as the concentration of Cu^2+^ is increased, the dimeric state of HL needs to be broken and the formed monomeric species then react with Cu^2+^ because of which there is a deviation from Beer–Lamberts law in the system. Further, the results from mass spectrometry suggested that the stoichiometry of the complex formed is by refluxing the reactants in appropriate ratios (2 : 1, ligand : metal). Also, we have tried to plot the Jobs plot for the system which was suggesting that the complex formed in the system is not 1 : 1, further supporting the mass spectrometric data.

**Fig. 10:**
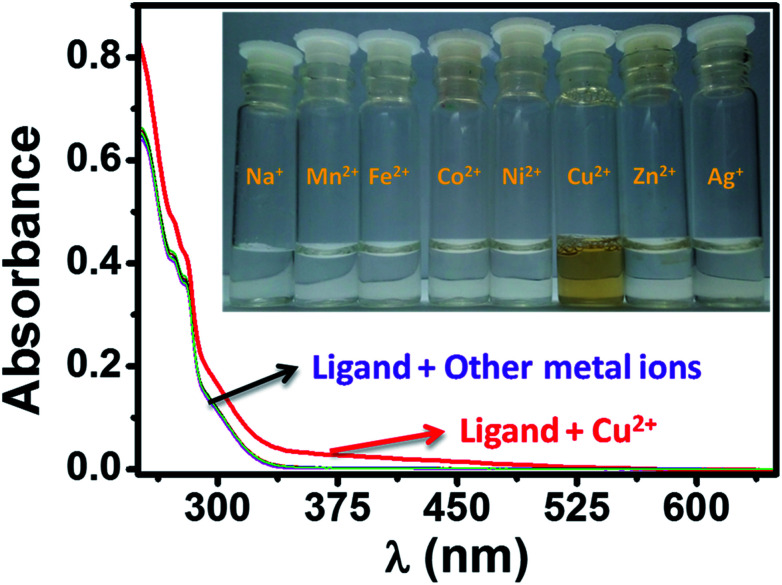
The UV-visible spectra of HL in the presence of two equivalents of Cu^2+^ (red trace) and in the presence of other metal ions. The inset picture showing the color change during reaction of HL with Cu^2+^ ion.

The selectivity of HL towards Cu^2+^ ions (red trace) and no such distinguishable changes could be observed during the reaction of HL with other tested metal ions (Fig. 10). The phenomenon is more evident from the picture taken from the solutions of HL and target metal ions. We could see immediate brown colorization of the solution only in case of Cu^2+^ ions. We also estimated by UV-visible studies, the detection limit of HL for Cu^2+^ was found to be 1.6 μM, which is much lower than the recommended value (31.5 μM) of the World Health Organization (WHO) in drinking water.

## Conclusions

To sum up, a Schiff base ligand, 3-((3-methoxybenzylidene)amino)-1*H*-pyrazol-5-ol, HL and its Cu(ii) complexes, 1 and 2 were synthesized and characterized. This study has presented a colorimetric sensor for Cu^2+^ ion detection. The electronic absorption studies shown that the detection limit of novel ligand, HL for Cu^2+^ ion was found to be 1.6 μM, which is much lower than the recommended value (31.5 μM) of the World Health Organization in drinking water, thus, can find significant application in the field work as a promising probe. According to MEP and ALIE surfaces HL has several important reactive molecular sites sensitive to electrophilic attacks. The lowest ALIE values were ∼188 kcal mol^−1^ and this descriptor predicted more reactive sites. Fukui *f*^+^ function of HL indicates that electron density increases in the near vicinity of C9–H17 bond, while Fukui *f*^−^ function indicates that electron density decreases in the near vicinity of C1–C2 bond. H-BDE descriptor indicates very high sensitivity of HL to autoxidation mechanism.

## Conflicts of interest

The authors have no conflicts to declare.

## Supplementary Material

RA-008-C8RA02905A-s001
