# Supplementary material for: Remarkable colorimetric sensing behavior of pyrazole-based chemosensor towards Cu(ii) ion detection: synthesis, characterization and theoretical investigations
Source: RSC Adv. 2018 May 17;8(32):18023–9. doi: 10.1039/c8ra02905a (PMC9080470; doi:10.1039/c8ra02905a)
Supplement: RA-008-C8RA02905A-s001 [file RA-008-C8RA02905A-s001.pdf]

Electronic Supplementary Material (ESI) for RSC Advances.
This journal is © The Royal Society of Chemistry 2018

Supporting Information

Remarkable Colorimetric Sensing Behavior of Pyrazole-based Chemosensor Towards Cu(II) Ion Detection: Synthesis, Characterization and Theoretical Investigations

Nagaraj Nayak,[a] Kollur Shiva Prasad,*,[a] Renjith Raveendran Pillai,[b] Stevan Armaković,[c] and Sanja J. Armaković[d]

[a] Chemistry Group, Manipal Centre for Natural Sciences, Manipal Academy of Higher Education, Manipal, Karnataka – 576 104, India.
[b] Department of Physics, TKM College of Arts and Science, Karicode, Kollam, Kerala, India.
[c] University of Novi Sad, Faculty of Sciences, Department of Physics, Trg D. Obradovića 4, 21000 Novi Sad, Serbia.
[d] University of Novi Sad, Faculty of Sciences, Department of Chemistry, Biochemistry and Environmental Protection, Trg D. Obradovića 3, 21000 Novi Sad, Serbia.

Contents

Figure S1: The FT-IR spectra of the **HL** and Cu(II) complexes, **1** and **2**.

Figure S2: The ^{13}C NMR spectrum of compound **HL**.

Figure S3: ESI-HRMS of complex **1**.

Figure S4: ESI-HRMS of complex **2**.

Table S1: Detection limit towards Cu^{2+} ion sensing by various chemosensors.

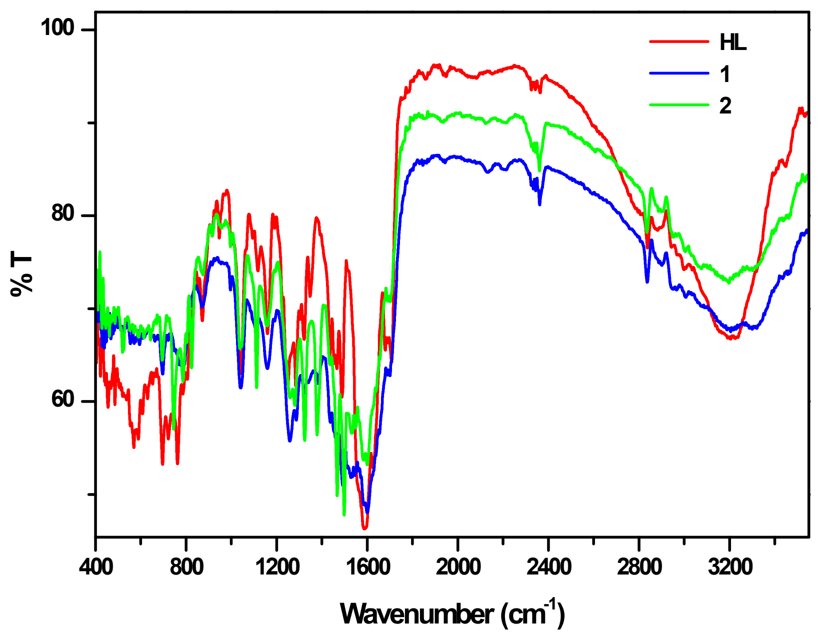

Figure S1: The FT-IR spectra of the **HL** and Cu(II) complexes, **1** and **2**.

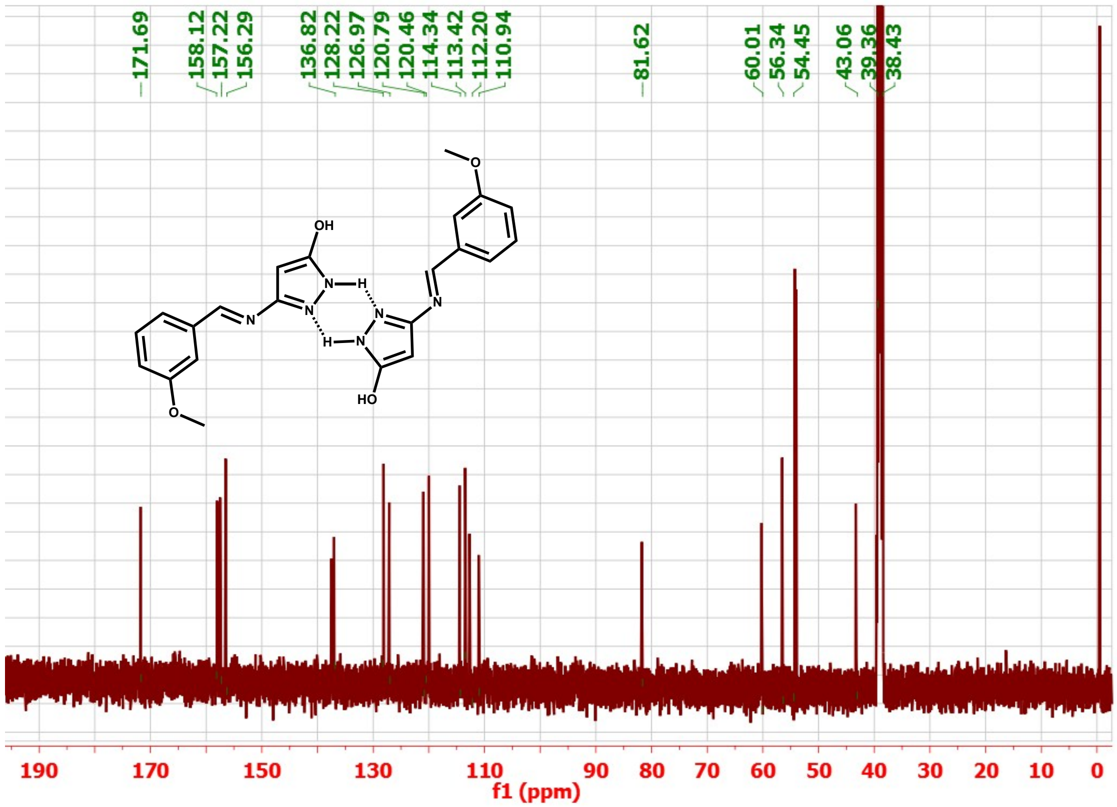

Figure S2: The ^{13}C NMR spectrum of compound **HL**.

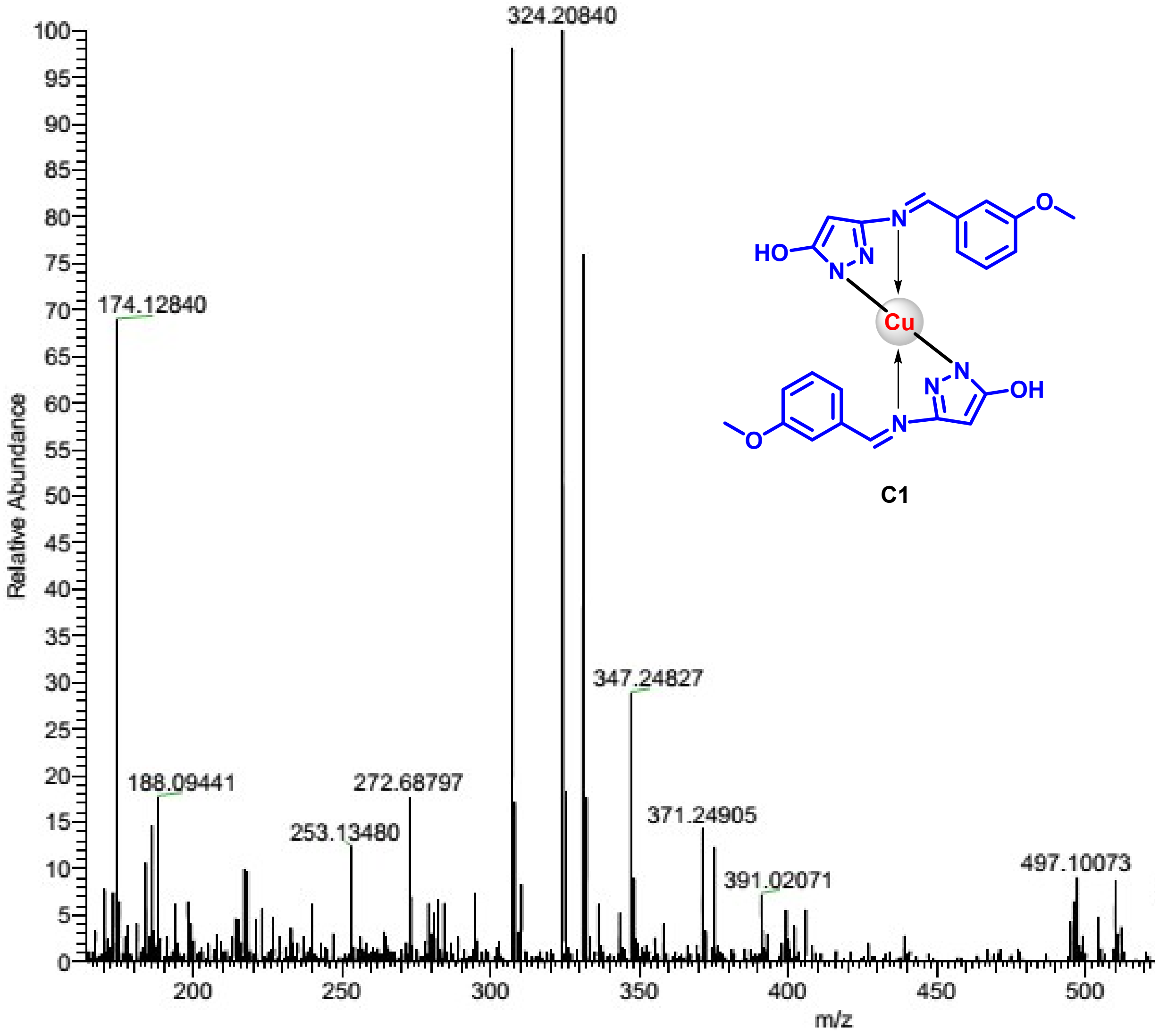

Figure S3: ESI-HRMS of complex **1**.

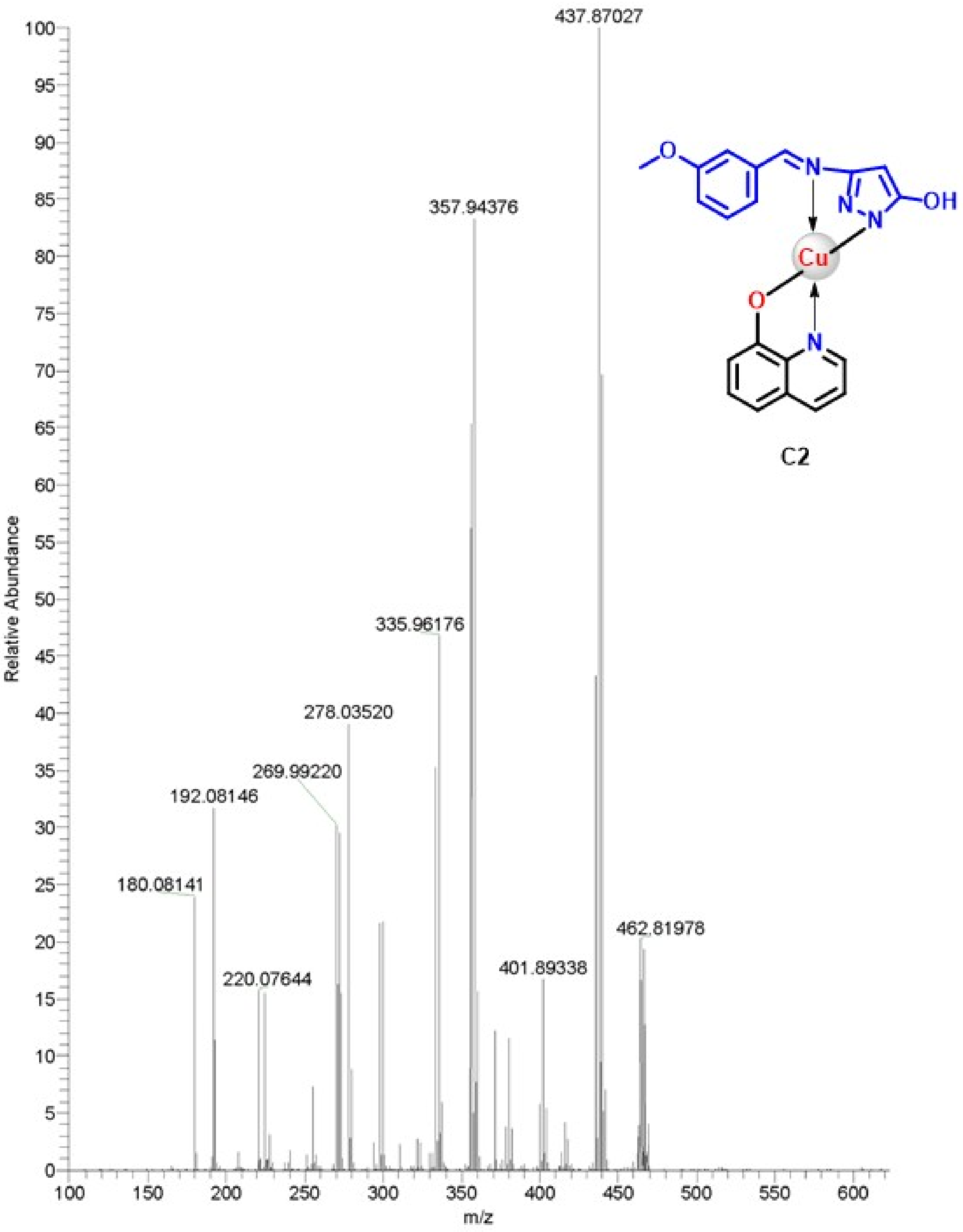

Figure S4: ESI-HRMS of complex **2**.

Table S1: Detection limit towards Cu^{2+} ion sensing by various chemosensors.

Sensor reported in the literature	Lowest detecting ability of Cu^{2+} ions from solution
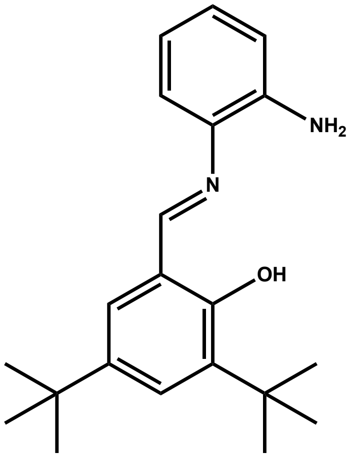 **Colorimetric sensor** doi: 10.1080/00958972.2017.1420787	1.66 μM
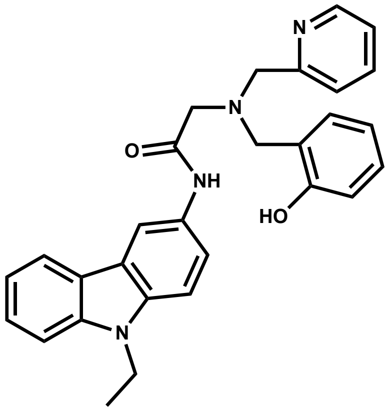 **Colorimetric sensor** doi: 10.1016/j.snb.2016.01.133	2.9 μM
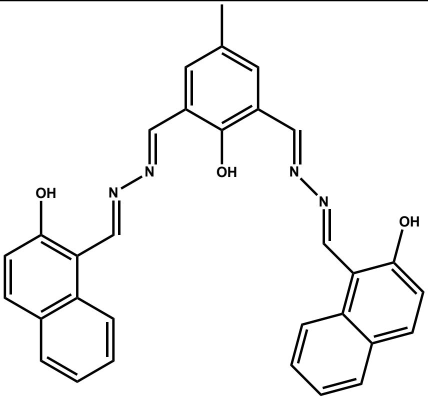 **Colorimetric sensor** doi: 10.1016/j.tetlet.2013.09.126	5 μM

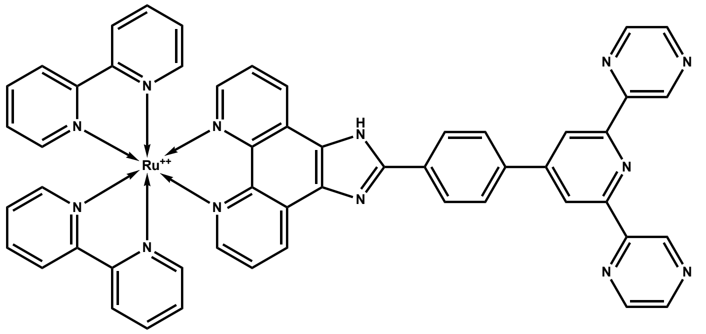<br>**Fluorescence sensor**<br>doi: 10.1016/j.snb.2015.06.124	2.73 µM
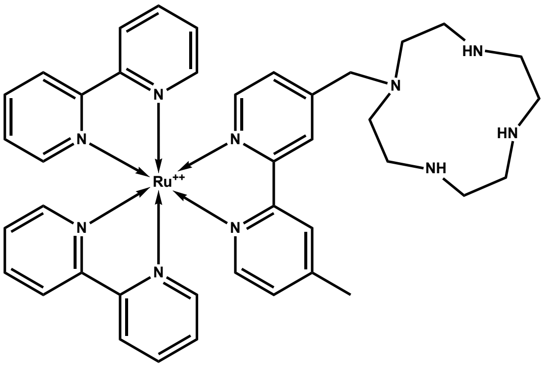<br>**Fluorescence sensor**<br>doi:10.1039/C3DT51047F	5.4 µM
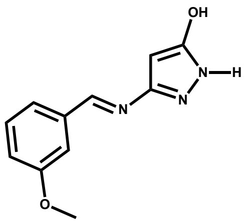<br>**Our system**	1.60 µM
